# Using body size as an indicator for age structure in field populations of *Aedes* *aegypti* (Diptera: Culicidae)

**DOI:** 10.1186/s13071-022-05605-z

**Published:** 2022-12-22

**Authors:** Eileen H. Jeffrey Gutiérrez, M. A. Riehle, K. R. Walker, K. C. Ernst, G. Davidowitz

**Affiliations:** 1 Graduate Interdisciplinary Program in Entomology and Insect Science, University of Arizona, 1140 E South Campus Drive, Forbes 410, Tucson, AZ 85721-0036 USA; 2 Dept. of Epidemiology and Biostatistics, College of Public Health, University of Arizona, 1295 N. Martin Ave., PO Box 245210, Tucson, AZ 85724 USA; 3 Dept. of Epidemiology and Biostatistics, School of Public Health, University of California, Berkeley, 2121 Berkeley Way, 94720-7360 Berkeley, USA

**Keywords:** *Aedesaegypti*, Dengue, Survival, Age, Surveillance, Vectors, Body size

## Abstract

**Background:**

The *Aedes* *aegypti* mosquito is a vector of several viruses including dengue, chikungunya, zika, and yellow fever. Vector surveillance and control are the primary methods used for the control and prevention of disease transmission; however, public health institutions largely rely on measures of population abundance as a trigger for initiating control activities. Previous research found evidence that at the northern edge of *Ae.* *aegypti*’s geographic range, survival, rather than abundance, is likely to be the factor limiting disease transmission. In this study, we sought to test the utility of using body size as an entomological index to surveil changes in the age structure of field-collected female *Aedes* *aegypti*.

**Methods:**

We collected female *Ae.* *aegypti* mosquitoes using BG sentinel traps in three cities at the northern edge of their geographic range. Collections took place during their active season over the course of 3 years. Female wing size was measured as an estimate of body size, and reproductive status was characterized by examining ovary tracheation. Chronological age was determined by measuring transcript abundance of an age-dependent gene. These data were then tested with female abundance at each site and weather data from the estimated larval development period and adulthood (1 week prior to capture). Two sources of weather data were tested to determine which was more appropriate for evaluating impacts on mosquito physiology. All variables were then used to parameterize structural equation models to predict age.

**Results:**

In comparing city-specific NOAA weather data and site-specific data from HOBO remote temperature and humidity loggers, we found that HOBO data were more tightly associated with body size. This information is useful for justifying the cost of more precise weather monitoring when studying intra-population heterogeneity of eco-physiological factors. We found that body size itself was not significantly associated with age. Of all the variables measured, we found that best fitting model for age included temperature during development, body size, female abundance, and relative humidity in the 1 week prior to capture . The strength of models improved drastically when testing one city at a time, with Hermosillo (the only study city with seasonal dengue transmission) having the best fitting model for age. Despite our finding that there was a bias in the body size of mosquitoes collected alive from the BG sentinel traps that favored large females, there was still sufficient variation in the size of females collected alive to show that inclusion of this entomological indicator improved the predictive capacity of our models.

**Conclusions:**

Inclusion of body size data increased the strength of weather-based models for age. Importantly, we found that variation in age was greater within cities than between cities, suggesting that modeling of age must be made on a city-by-city basis. These results contribute to efforts to use weather forecasts to predict changes in the probability of disease transmission by mosquito vectors.

**Graphical abstract:**

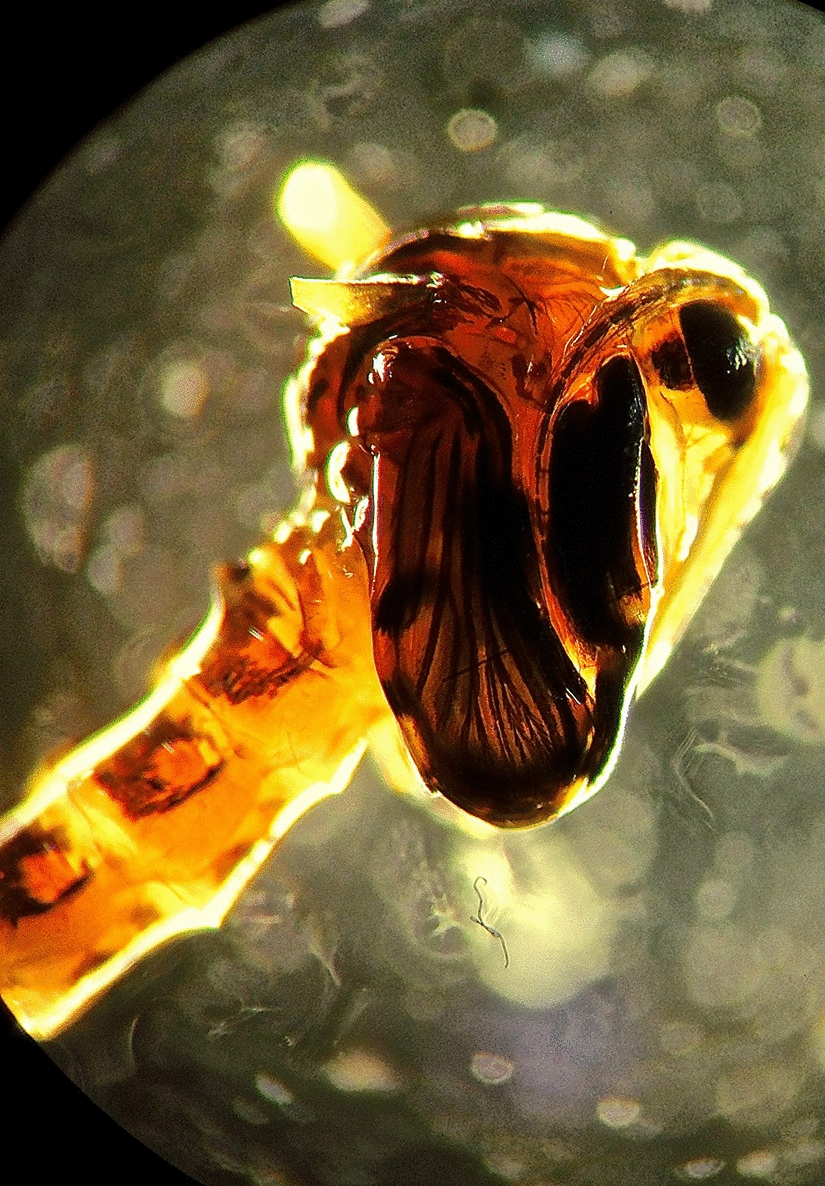

**Supplementary Information:**

The online version contains supplementary material available at 10.1186/s13071-022-05605-z.

## Background

The *Aedes* *aegypti* mosquito is a vector of several viruses including dengue, chikungunya, zika, and yellow fever. Of the arboviral diseases, dengue has the greatest overall impact due to its prevalence and immense economic burden [1, 2]. The global incidence of dengue fever has been increasing dramatically, more than doubling every 10 years, with nearly half of the world’s population at risk of infection [3]. Mosquito surveillance and control continue to be the most effective methods for regulating transmission due to the lack of available vaccines for *Aedes*-borne pathogens [3]. Indeed, arboviral infection may not confer lifelong immunity [4–6], and climatic warming in temperate areas is expected to increase arbovirus transmission risk by increasing environmental suitability for the persistence of vector populations [7, 8]. Changes in disease distribution are especially problematic because the introduction of disease into immunologically naive populations increases the intensity of pathogen replication and the severity of illness and infectiousness [9]. For these reasons, improved surveillance and control activities that reduce disease transmission are greatly needed.

There are several physiological and behavioral factors which regulate the likelihood that a mosquito will transmit disease, otherwise known as vectorial capacity. These factors include the mosquito’s survival rates, biting frequency, likelihood of encountering a human, reproductive rate, susceptibility to infection, and the incubation period of the pathogen [10]. Despite this host of factors, local public health authorities primarily rely on reports of increased vector abundance to determine whether surveillance and control efforts should be intensified. However, mosquito abundance is not always the primary factor limiting disease transmission as limiting factors will vary with the spatiotemporal range and distribution of the vector and pathogen. For example, despite abundant populations of *Ae.* *aegypti* within cities in the desert of the Sonoran Southwest in North America, local dengue transmission has been infrequent in Nogales, Sonora, Mexico and has not occurred at all in Tucson, Arizona, USA. Both cities engage in a significant amount of travel and trade with Hermosillo, Sonora, Mexico, which has regular seasonal dengue transmission. Furthermore, *Ae.* *aegypti* adult, larval, and pupal abundance was higher in Nogales compared to Hermosillo in 2013 (adult abundance was higher in Hermosillo in one of the 3 months of the study) [11] and the House Index (proportion of houses positive for larvae) was highest in Nogales, followed by Tucson, and lowest in Hermosillo [12], which begs the question, what is actually limiting dengue transmission in this region? It is of great public health interest to increase our understanding of how vector-borne disease transmission is regulated to identify appropriate surveillance methods for that area.

So while surveilling for increases in population abundance may work for anticipating disease risk in some areas [13, 14], this is clearly not the case for the Sonoran Southwest. It is also unlikely that genetic differences are responsible for the varying transmission rates in this region. Previous sampling of *Ae.* *aegypti* that included Nogales, Hermosillo, and Tucson found that all three cities’ populations belong to the same genetically distinct sub-group, differentiating them from other populations tested in Arizona, Texas, and Florida [15]. This is likely the result of frequent human trade and travel between locations. Following analysis of the differences in population density and age structure, Ernst et al. (2016) concluded that the stark difference in age structure of *Ae.* *aegypti* females between Nogales and Hermosillo was likely responsible for the difference in transmission dynamics and not abundance. Females in Hermosillo appeared to have consistently higher survival rates than those in Nogales. The survival rate of a female mosquito is an important factor in transmission potential because after consuming an infected blood meal, the female must outlive the incubation period of the virus before being able to transmit the pathogen [16, 17]. This means that only the oldest subset of a given population is capable of disease transmission. Unfortunately, testing individual field-collected mosquitoes for age to get an idea of survival rate is a costly and time-consuming process. Health departments are typically functioning under significant financial constraints and cannot incorporate costly new methods for vector surveillance into their control programs. Where survival is a limiting factor regulating dengue transmission, it would be extremely useful to identify a simple and sustainable proxy for characterizing age in field populations. While arboviral transmission can be affected by several other factors beyond abundance and survival, such as variation in host-vector interactions and in the extrinsic incubation period [18–20], in this study we focused on mosquito survival as it was identified as a transmission-limiting factor in Ernst et al. (2016).

One potential indicator for longevity in field mosquitoes is body size. Mosquitoes are holometabolous insects, which means that their size in adulthood is determined by environmental conditions during larval development. Once they emerge as adults, they will remain the same size throughout adulthood. The developmental conditions of a mosquito, such as food availability and temperature, have significant impacts on many of the physiological and behavioral components that determine vector capacity [21–24]. Beyond affecting adult body size, developmental conditions are known to alter resource allocation during adulthood into maintenance of the soma [25] or shifting prioritization among reproduction, immunity, and lifespan through changes in the regulation of several biochemical pathways [26–32]. For example, a large, well-provisioned female mosquito is more likely to invest a greater proportion of her blood meals into reproduction. Conversely, a small, ill-provisioned female is less likely to invest as much into reproduction and more likely to allocate incoming resources to self-maintenance [23, 32]. Recent work has provided mechanistic insights into how resource limitation and competition during larval stages can also modulate temperature-dependence of fitness in *Ae.* *aegypti*, demonstrating that spatiotemporal variation of resources among developmental sites can give rise to heterogeneity in fitness even within a population largely experiencing the same temperatures [34, 35].

Since mosquitoes are ectotherms, size is also determined by temperature during development [36, 37]. Warmer temperatures speed up the rate of development, reducing the time available for feeding, thereby producing smaller mosquitoes. The opposite is also true, where colder temperatures slow down the rate of development allowing more time for feeding and producing larger mosquitoes. This is an oversimplification of the mechanisms behind the temperature-size rule, the details of which have yet to be fully resolved; however, as discussed above, resource limitation during development can modulate temperature-dependent traits such as the growth rate, adult size, and fitness [34, 35]. As a consequence, size (in conjunction with temperature) can be used as an indicator for an insect’s life-history tradeoffs [32, 38, 39], in effect describing heterogeneity in longevity, survival and fecundity [38, 40–44]. Size can also impact desiccation resistance in mosquitoes [45–48], indirectly impacting survival and consequently age structure in conditions of low humidity [40, 49, 50]. Additionally, body size is negatively associated with biting frequency and dispersal distance, and positively associated with predation risk during immature and adult phases [51, 52], constituting additional mechanisms by which heterogeneity in size can impact disease risk [24, 53]. However, in the context of the Sonoran Desert region specifically, our interest was to investigate if body size in *Ae.* *aegypti* can be linked with differential mortality in areas at risk of emergent dengue transmission and thus potentially gain a valuable indicator for informing public health surveillance.

In this study, we tested whether variation in body size, female abundance, temperature, and/or humidity can predict age in field-collected, adult female *Ae.* *aegypti*. The cities in the study were sampled over a period of 3 years, have robust populations of *Ae.* *aegypti*, and have very different rates of local dengue transmission despite their geographic proximity. We also determined chronological age using a technique based on measuring the abundance of an age-dependent gene, SCP-1 [54, 55]. Contrary to the findings of several studies that have looked for associations among mosquito body size, age, and survival, we expected to find that the inclusion of measurements of size along with weather data would strengthen our ability to predict changes in population age structure. Previous field studies that tested the association among longevity, survival, and body size were based in tropical locations where temperature-mediated variation in size and longevity/survival would be minimal compared to locations at the northern edge of *Ae.* *aegypti’s* geographic range [53, 56]. We hypothesized that range-edge populations are distinct in the degree to which environmental conditions impact survival/longevity and consequently their ability to transmit disease.

## Methods

### Study area

**Fig. 1:**
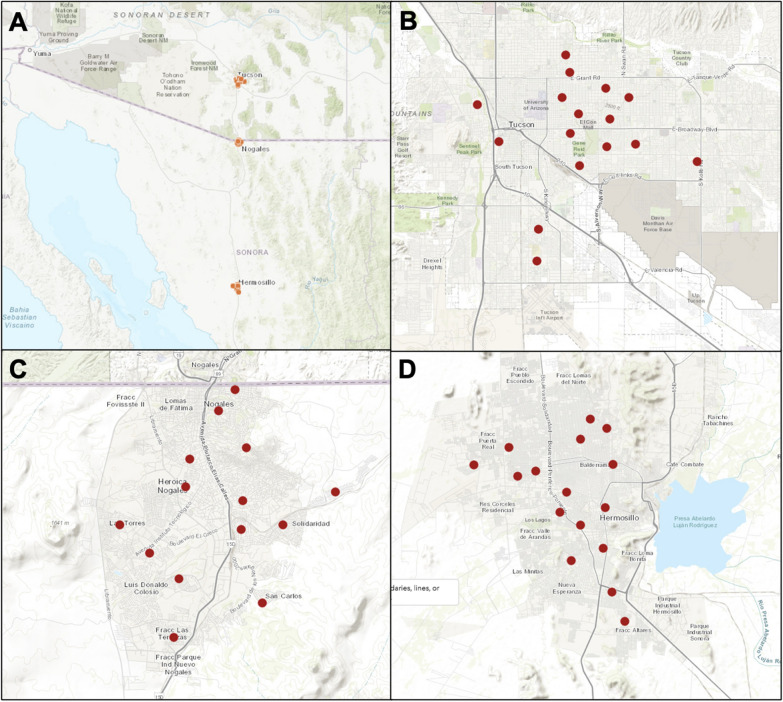
Study cities and their collection sites. **A** Study cities, Tucson, Nogales, and Hermosillo. **B** Collection sites within Tucson, Arizona. **C** Collection sites within Nogales, Sonora, Mexico. **D** Collection sites within Hermosillo, Sonora, Mexico

Adult mosquitoes were collected from households in three cities over a 4-day period, once a month. ArcGIS was used to generate random points at least 1 km away from each other to inform trap site selection; 500-m buffer zones were then generated around each point to guide selection of residences with snowball sampling through study affiliates as to where traps would be placed. Each city had between 15 and 40 trapping sites, depending on the year (Fig. 1; Additional file 4: Table S1). The study cities occupy a latitudinal transect at the northern edge of the geographic range of *Ae.* *aegypti* (Fig. 1a)*.* At the southern end of this transect is Hermosillo, Sonora, Mexico (29.0989°N, 110.9542°W), a city where the *Ae.* *aegypti* population has maintained local, seasonal transmission of the dengue viruses. At the center of the transect is Nogales, Sonora, Mexico (31.1907°N, 110.5645°W), which saw its first cases of local transmission in 2014, during the study period. At the northern end of this transect is Tucson, Arizona, USA (32.2217°N, 110.9264°W), which has no documented cases of locally acquired dengue fever before or during the study period. This transect of the Sonoran Desert occupies 394.2 km and a ranges in elevation from 210 m (Hermosillo, Mexico) above sea level to 1199 m (Nogales, Mexico). Collections were limited to the 3 months of the monsoon season, July, August, and September (sometimes in October), because of the significant seasonal increase in mosquito abundance following summer monsoon precipitation events and subsequent dengue transmission (Additional file 4: Table S2).

### Mosquito sampling and testing for bias

Biogents (BG) Sentinel traps were baited with octanol and lactic acid lures and were connected to either a battery or a household electric supply. BG Sentinel traps have been found to be about as efficient as human landing rate or backpack aspirators and more efficient than oviposition traps for evaluating abundance in the field [57–59]. BG Sentinel traps have a slight bias for host-seeking *Ae.* *aegypti* females, and the location of the trap is a potential source of bias against nulliparous females [60]. Also, adult mosquito abundance is not affected by the use of insecticides indoors [61]. In essence, known trapping biases would result in over-sampling of our target group of blood-fed females, which, while preferable for public health surveillance, is not optimal for our purposes of sampling females of a range of ages.

Adults collected from traps were aspirated into containers, taken to locations within each city for quantification, then transported to the central laboratory in Tucson for analysis. Dead adult females were counted and included in abundance data but not included in any parity or age analyses. Live females were stored in a – 80 ºC freezer until processing (*N* = 3920 measures of individual size and *N* = 4739 individuals analyzed for parity status). Since traps were only checked once a day during the collection periods, some mortality occurred in the field-collected females that could have caused a bias in the body size of the surviving females. To test if there was a size bias due to differential survival in the trap, dead females from Tucson (*N* = 60) were measured and compared to live females (*N* = 78) from the same subset of sites and months. Dead females from Nogales and Hermosillo were discarded and unable to be analyzed for this purpose.

### Mosquito age and parity assessments

Ovaries were dissected to determine parity. Visual inspection of trachea in the ovaries allowed us to determine whether a female had completed a reproductive cycle or not [62, 63]. Tracheae that are tightly coiled are considered nulliparous, having never completed a reproductive cycle. Individuals with extended tracheae are considered parous, since once the tracheae extend to transport oxygen to developing eggs they will not recoil. Individuals determined to have completed a reproductive cycle, and/or had a visible, undigested blood meal, and/or eggs were all considered as parous. Parity serves as a physiological marker of age and for observing changes over time in biting persistence and the human-to-mosquito contact rate for a given location [63–65]. In this study we characterized females with a visible bloodmeal as parous; therefore, it is more accurate to consider parity in this context as a measure of the percent of blood-fed females in a population.

Classification of individuals into age groups was done with a genomic age-grading technique using real-time PCR assays of an age-dependent gene, SCP-1 [54]. SCP-1 reliably decreases in expression as a mosquito ages, allowing for age to be inferred based upon the abundance of SCP-1 gene transcripts as compared to transcript titers of a control, RPS17. Females tested for age are classified categorically as being either 0–5, 6–14, or ≥ 15 days old with an accuracy of 90%. Host seeking in *Ae.* *aegypti* females begins at 36–48 h post-emergence [66], and the average extrinsic incubation period of the dengue virus ranges from 6.5 to 15 days [17]. This means that the youngest age group cannot transmit disease while the 6–14-day-old group can but is unlikely to include possible vectors, and the oldest age group will consist of likely more competent vectors. These age groups were used to get a general estimate of when the developmental period occurred and the corresponding average temperature and relative humidity data for that date range. In addition, a continuous measure of age was adapted from the transcript abundance values of SCP-1 using data from [54] and was used in the regression models. To accomplish this, we related SCP-1 expression levels to log-transformed ages (R package rms; Harrell 2016) using an ordinary least squares (OLS) regression model on laboratory and semi-field reared mosquitoes with known ages. The resulting non-linear relationship was then modeled using a three-knot restricted cubic spline. The relationship between transcript abundance of SCP and chronological age was not significantly different between fed and unfed mosquitoes. This method for obtaining a continuous measure of age was used in a recent study which compared the accuracy of this age-grading method to that of using near-infrared spectroscopy (NIRS) and found that NIRS was not as reliable as SCP-1 transcript expression [67]. The R code used to generate this continuous age variable can be found in the https://github.com/ejeffreygutierrez/Predicting-Aedes-age.

### Wing measurements and weather data

Wings were removed from field-collected females and affixed onto glass microscope slides with a drop of water. Samples were secured onto the slides with a glass cover slip fixed with tape on the sides. Length was measured along the major axis, from the proximal to the distal end for each wing, as described in [68]. In a previous publication by this study’s authors, wing length of the major axis was found to be more tightly associated with age at death than wing area or length of the minor axis [69].

Seven-day averages of temperature, diurnal temperature range, average daily maximum and minimum temperature, and percent relative humidity were estimated using city-specific, historical weather data from the National Oceanic and Atmospheric Association (NOAA) and using site-specific (sites within cities) averages from remote climate loggers (HOBO Pro v2, Onset). Weather averages from NOAA and the HOBOs were each tested against wing length to determine which data source was a better fit. Although female mosquitoes collected at the same time, of the same age group, are still likely to have variation in development time, it is not currently possible to estimate development time in field-collected mosquitoes without information on resource availability within their specific development site. For this reason, we chose to use 7-day averages for estimating average developmental temperature. We previously found an average developmental period of about eight days for nutritionally-stressed *Ae.* *aegypti*  derived from eggs collected from three locations in Tucson, Arizona, and inclusion of the length of the developmental period did not improve models for estimating age at death [69]. The R code used to generate averages of climate variables from the raw HOBO and NOAA data can be found in  Supp. Mat. SM1.

Using our sample-specific age data, we also tested a new technique for estimating when a particular female developed. This technique involves back-casting by different periods of time starting from the date a sample was captured, based on the results of the age-dependent gene expression analyses (Additional file 4: Table S3). Estimating the developmental period of individual mosquitoes to study the impact of environmental factors on adult longevity is a novel approach, considering previous studies typically assign the same estimated development period to all mosquitoes sampled [70].

A number of dates are missing HOBO data: Nogales 2013 August, Age group 1 was generated from 6 days (missing one day of weather data). No HOBO data exist for Hermosillo 2015 or August 2013, Nogales in August of 2013 for age groups 2 and 3, or July 2013 for any city. This lack of data due to missing or defective HOBOs reduced the total N in our path analyses from 3920 to 1125.

### Statistical analysis

All data were analyzed on R 1.0.143 [71] and JMP [72]. ANOVA and linear regression were used to test the impact of the explanatory variables: parity, temperature during development, wing length, female abundance, and relative humidity in the 1 week prior to capture and temperature in the 1 week prior to capture on the response variable, age. The R code used in these analyses can be found in Supp. Mat. SM1.

To test direct and indirect effects of explanatory factors on wing length and age at death, we used a combination of factor analysis and regression analysis known as multivariate path analysis or structural equation modeling (SEM). R was used for the SEM using the variables average temperature during development, wing length, female abundance, relative humidity in the 1 week prior to capture and temperature in the 1 week prior to capture, and age. Supporting materials for these analyses are in Additional file 4: Tables S4 and S5 as recommended by best practices for reporting results of SEMs [73]. These models were fitted to the explanatory variables listed above using the R packages car, QuantPsyc, ggm, semPlot, lavaan, nlme, and devtools.

The strengths of the models tested were first evaluated by comparing their AIC values, which consider indirect effects and prioritize simplicity in model selection by imposing a penalty for each additional variable used. Models with the lowest AIC scores were then evaluated by their adjusted *R*^2^ to determine goodness of fit.

## Results

The wing lengths of field-collected females were tested for normality, and their residuals were found to be non-normally distributed (Shapiro-Wilk test; *P* < 0.01, *N* = 138). Wing length was log transformed and tested again but remained non-normally distributed (Shapiro-Wilk test; *P* < 0.01, *N* = 138). City-specific wing length averages and standard deviations are reported in Additional file 4: Table S4. Distributions of wing length for each year-city-month and corresponding results of the Shapiro-Wilk test are shown in Additional file 1: Fig. S1. These distributions show that departures from normality are modest. A piecewise structural equation model was used to account for the non-normally distributed data.

### Size bias associated with trapping method/collection times

**Fig. 2:**
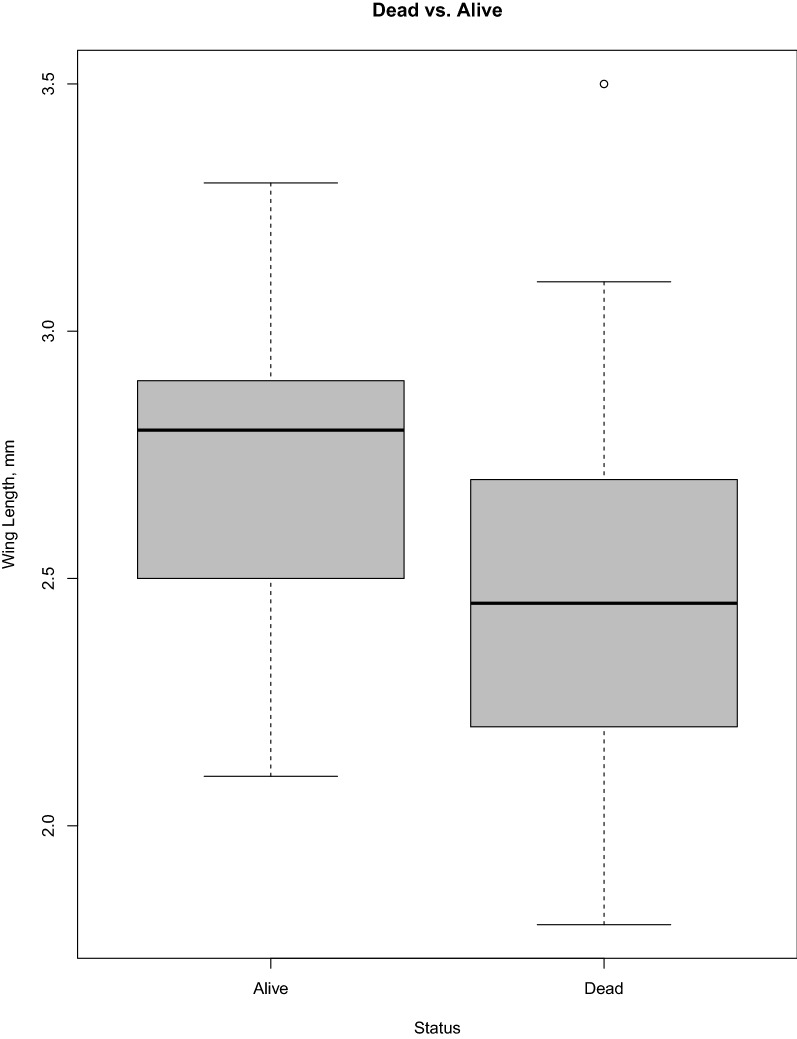
Average wing size of dead vs. alive females. Mean wing length of dead and alive females from four sites and 3 months, Tucson 2014. Comparison of means using ANOVA and Turkey-Kramer HSD found that there was a significant difference with dead females being significantly smaller than those collected alive (*N* = 137)

A Student’s paired t-test of females that were dead vs. alive showed that smaller females were more likely than larger females to have perished in the traps before being collected, *t* = 5.14, *df* = 136, *P* < 0.0001 (Fig. 2). Females collected alive and used for parity and age testing had a mean wing length of 2.73 mm, and those that were dead upon collection and unable to be used for further analysis had an average wing length of 2.47 mm.

### HOBO vs. NOAA weather data

**Fig. 3:**
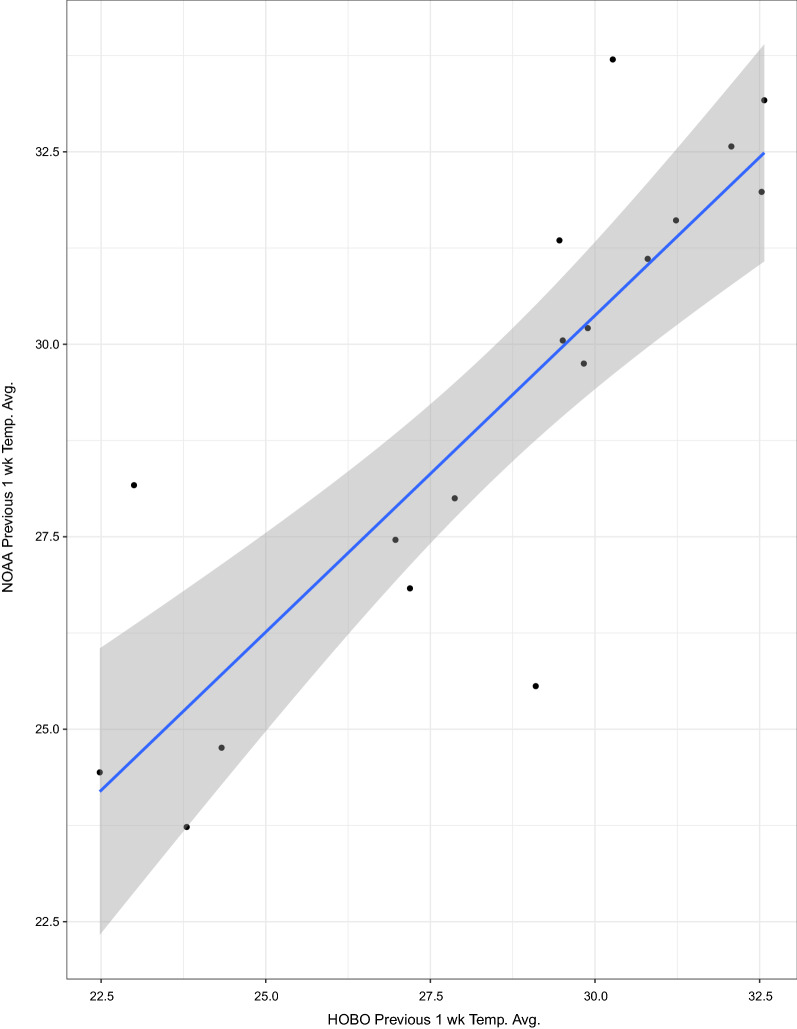
NOAA vs. HOBO temperature averages. We compared both sources of data from the week prior to collection to show the nature of the bias of the more generalized data source (NOAA). NOAA’s 1-week temperature averages are significantly higher than HOBO averages, making HOBO data more accurate for predictions regarding the physiological responses of mosquitoes to weather variability

Linear regression analysis of average temperature during development using HOBO data and NOAA data showed that NOAA data overestimate the site-specific HOBO data (Fig. 3). Weather averages from both sources were also regressed against wing length to see which was more closely associated with length and by proxy, which is more closely associated with conditions experienced by larvae during development. HOBO data produced the strongest model for length (Table 1a).

**Table 1:** Comparing AIC values between models

Explanatory variables	NOAA	HOBO
**A. Models for wing length**		
Avg. Dev. Temp., Prev. 1wk. RH, Prev. 1wk Temp	439.596*	413.4185*
Avg. Dev. Temp., Prev. 1wk. RH	525.7594	415.9599
Prev. 1wk. RH, Prev. 1wk Temp	729.2559	668.7226
Avg. Dev. Temp., Prev. 1wk. Temp	440.2945	421.3252
Avg. Dev. Temp., Prev. 1wk. RH, Prev. 1wk Temp., # females/site	441.444	423.298
Avg. Dev. Temp., Prev. 1wk. RH., # females/site	527.7311	427.8371
Prev. 1wk. RH, Prev. 1wk Temp., # females/site	566.2172	680.3344
Avg. Dev. Temp., Prev. 1wk. Temp., # females/site	442.3052	433.7088
**B. Models for age**		
Avg. Dev. Temp., Prev. 1wk. RH, Prev. 1wk Temp	9818.04	8641.58
Avg. Dev. Temp., Prev. 1wk. RH	10,791.66	8639.58
Prev. 1wk. RH, Length	8555.46	7097.97
Avg. Dev. Temp., Prev. 1wk. RH, Length	8553.65	6847.56*
Avg. Dev. Temp., Prev. 1wk. RH, Length, # females/site	8555.00	6848.57*
Avg. Dev. Temp., Prev. 1wk. RH, # females/site	10,793.58	8641.21
Avg. Dev. Temp., Prev. 1wk. RH, Prev. 1wk Temp, # females/site	9820.04	8643.16
Avg. Dev. Temp., Prev. 1wk. RH, Prev. 1wk Temp, # females/site, Length	7762.23	6850.29
Avg. Dev. Temp., Prev. 1wk. RH, Prev. 1wk Temp., Length	7760.67*	6849.47

Asterisks denote the lowest AIC values

### Wing length and temperature during development

**Fig. 4:**
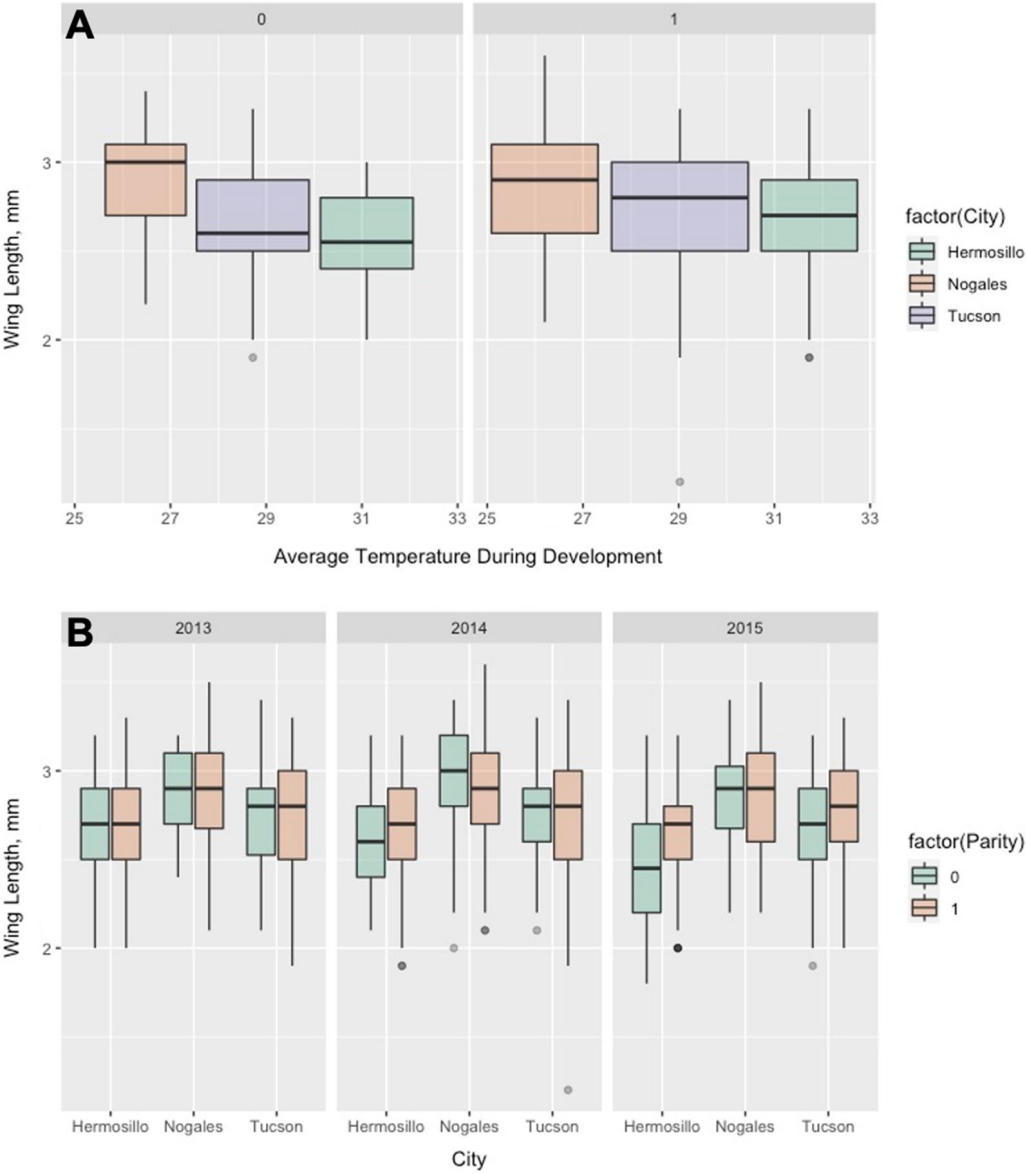
Wing length among cities, by parity status. **A** By temperature during development. **B** By year

Linear regression analyses of wing length and average temperature during development using HOBO data showed that statistical significance improved when analyzing females separately by their parity status. Nulliparous females had a stronger relationship between wing length and average temperature during development compared to parous females (Fig. 4a). Nulliparous; Adj. *r*^2^ = 0.134, RMSE = 0.298, *df* = 163, *P* < 0.0001. Parous; Adj. *r*^2^ = 0.045, RMSE = 0.287, *df* = 946. All females; Adj. *r*^2^ = 0.055, RMSE = 0.291, *df* = 1119, *P* < 0.0001.

Repeating the same analyses using NOAA data also showed that wing length and average temperature during development are more closely related in nulliparous females. Nulliparous; Adj. *r*^2^ = 0.116, RMSE = 0.306, *df* = 215, *P* < 0.0001. Parous; Adj. *r*^2^ = 0.058, RMSE = 0.285, *df* = 1289. All females; Adj. *r*^2^ = 0.067, RMSE = 0.291, *df* = 1526, *P* < 0.0001.

### Wing length and age

We used linear regression to test whether length was associated with age and found no statistical significance, Adj. *r*^2^ = 0.0002, RMSE = 5.274, *df* = 1545, *P* = 0.253. We then tested each city individually; the results were also not significant; Hermosillo: Adj. *r*^2^ = − 0.002, RMSE = 5.317, *df* = 521, *P* = 0.949. Nogales: Adj. *r*^2^ = − 0.002, RMSE = 4.89, *df* = 374, *P* = 0.643. Tucson: Adj. *r*^2^ = 0.000, RMSE = 5.41, *df* = 648, *P* = 0.258.

### Path analysis for wing length

**Fig. 5:**
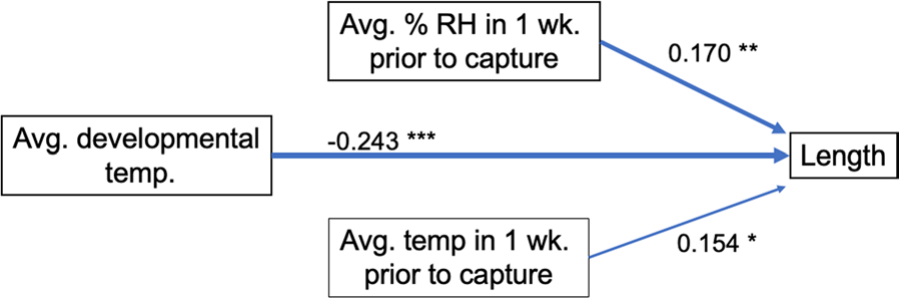
Path analysis for wing length. The model for predicting wing length that had the lowest AIC value used HOBO data and included avg. temp. during development, and avg. relative humidity and temp. in the 1 week prior to capture. Path values are standardized regression coefficients. Solid lines signify significant relationships; dashed lines are insignificant pathways which were omitted from the final analyses. *P < 0.05, **P < 0.01, ***P < 0.001

A path analysis for wing length using temperature during development, temperature 1 week prior to collection, and percent relative humidity in the 1 week prior to collection showed that all three variables had a significant effect on wing length (Fig. 5). This model for predicting length had the lowest AIC score compared to other models tested, which included female abundance (Table 1a). It should be noted that there is an overlap of 3 days included in the average temperature during development and in the average temperature 1 week prior to collection for the youngest age group.

### Path analysis for age

**Fig. 6:**
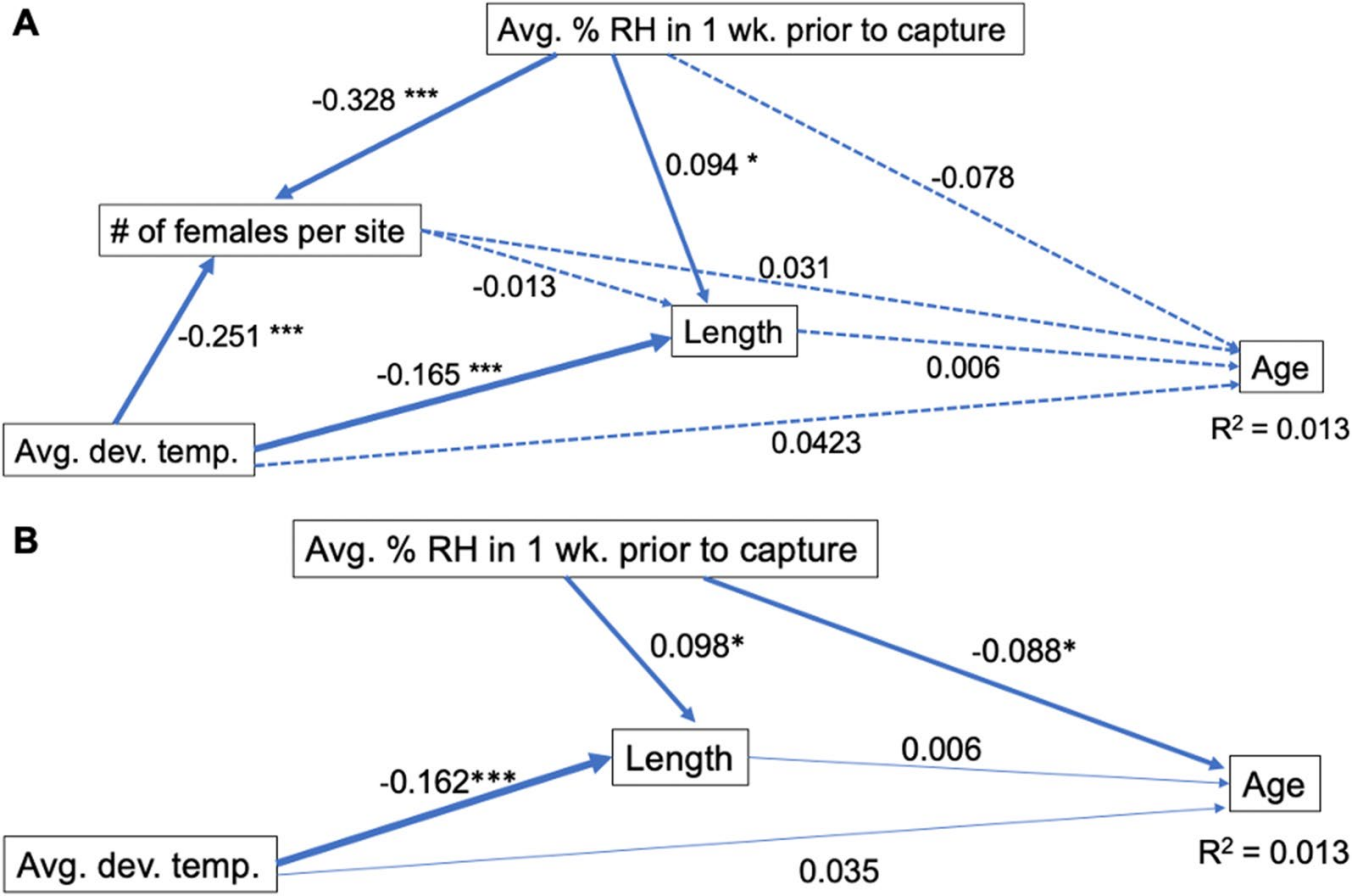
Path analysis for age, all cities. **A** Model for predicting age using all variables. **B** The model for predicting age that had the lowest AIC score and included avg. temp. during development, wing length, and avg. relative humidity in the 1wk prior to collection. Both models had *r*^2^ of 0.013. Path values are standardized regression coefficients. Solid lines signify significant relationships; dashed lines are pathways with insignificant p values. *P < 0.05, **P < 0.01, ***P < 0.001

**Fig. 7:**
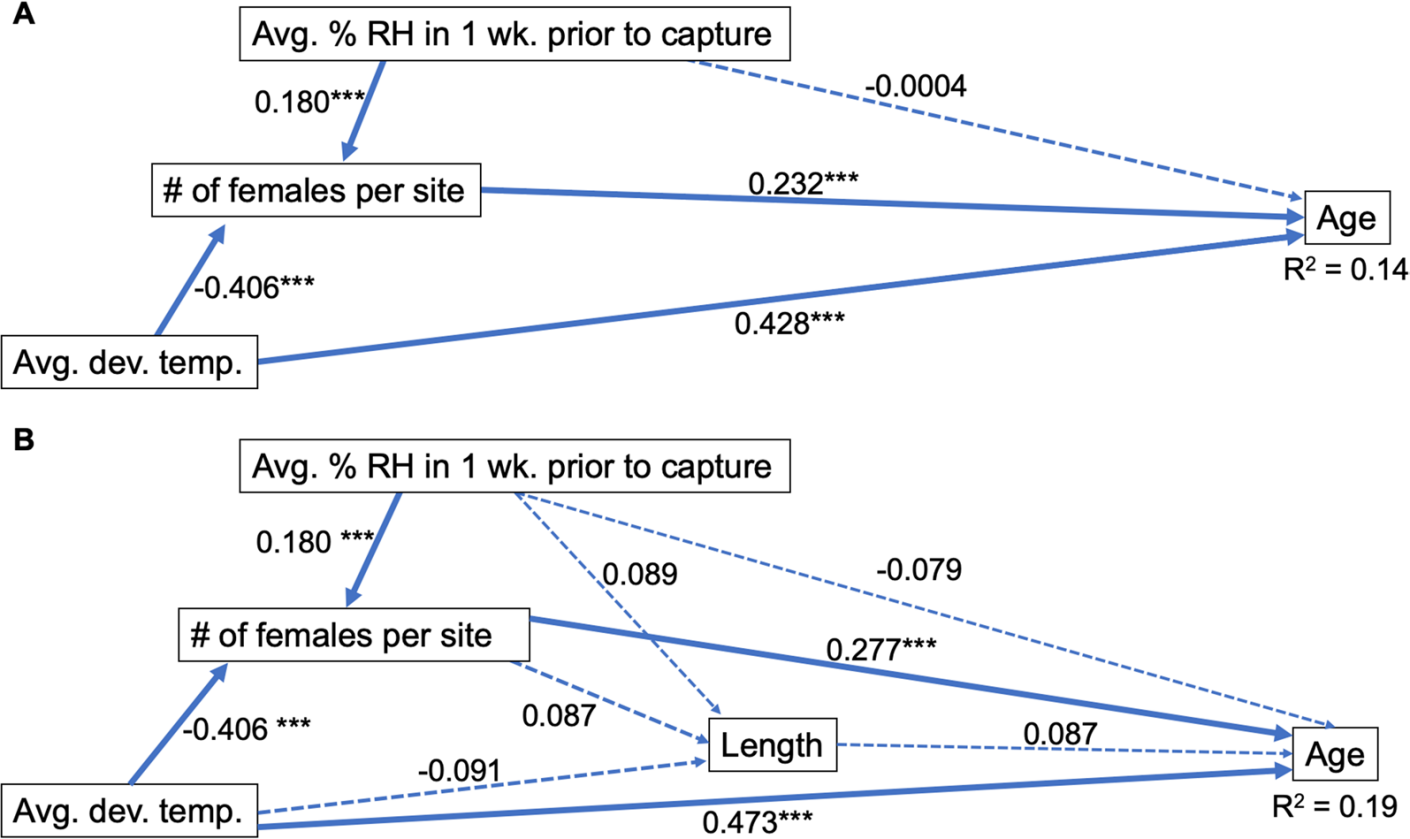
Path analysis for age, Hermosillo only. City-specific models for age prediction were more robust than the model including all cities. **A** Model for age without accounting for wing length has an *r*^2^ of 0.14. **B** The strongest model for predicting age included wing length and had an *r*^2^ of 0.19 (Hermosillo). Path values are standardized regression coefficients. Solid lines signify significant relationships; dashed lines are insignificant pathways that were omitted from the final analyses. *P < 0.05, **P < 0.01, ***P < 0.001

The sets of variables used for constructing the path analysis for testing direct and indirect effects on age for all three cities was selected by exploring the two sets of variables that produced the strongest models based on AIC scores since they were within 0.01% difference in AIC value (Table 1b). One of these models used HOBO weather averages and had an AIC of 6847.56 using temperature during development, percent relative humidity in the 1 week prior to collection, and wing length, had an *r*^2^ of 0.013, and excluded the variables female abundance and temperature 1 week prior to collection (Fig. 6a). The next best model had an AIC value of 6848.57 and was only different in that it included female abundance (Fig. 6b)*.* Applying these models for each city separately proved to be much more effective for predicting age with Hermosillo being most robust with an AIC of 1622.46 and an *r*^2^ of 0.19 (Table 2). Including length and female abundance together increased predictive capacity by more than their individual *r*^2^ added together (Fig. 7a, b).

**Table 2:** Comparing AIC values between models for each city

Explanatory variables	Statistic	Tucson	Nogales	Hermosillo
Avg. Dev. Temp., Prev. 1wk. RH	AIC	4372.63	2267.43	2005.72
	*df*	718	384	319
	Adj. *r*^2^	0.07	0.07	0.10
Avg. Female Abundance, Avg. Dev. Temp., Prev. 1wk. RH	AIC	4373.57	2269.05	1993.03
	*df*	717	383	318
	Adj. *r*^2^	0.07	0.08	0.14
Avg. Dev. Temp., Prev. 1wk. RH, Length	AIC	3367.93	1845.70	1622.46
	*df*	550	310	260
	Adj. *r*^2^	0.08	0.08	0.13
Avg. Female Abundance, Avg. Dev. Temp., Prev. 1wk. RH, Length	AIC	3368.81	1847.72	1607.1*
	*df*	549	309	259
	Adj. *r*^2^	0.08	0.07	0.19

AIC values and *r*^2^ improved when testing within cities. Asterisk denotes the lowest AIC value

## Discussion

In this study, we aimed to use a combination of weather variables, site-specific female abundance, and measurements of wing length to explain observed variation in age in the field. We found that for the three cities sampled, the best variables for modeling age were average temperature during development, female abundance, wing length, and average relative humidity in the 1 week prior to capture. Although combining all three cities in the model provided the greatest range in temperature and relative humidity, we found that replicating this model for each city individually produced stronger models for age. This is likely because the variation in age within cities was greater than the variation among cities compared to wing length which has more inter-city variation (Additional file 2: Fig. S2, Additional file 3: Fig. S3), as evidenced by the general agreement between the (inter- and intra-city) NOAA and HOBO-derived models for wing length and then the significant disagreement between those models when predicting age. Interactions between temperature and spatiotemporal variation in resource availability could explain why our predictors for age perform differently depending on the city [34, 35].

Of the city-specific models, Hermosillo (the only city with regular dengue transmission) produced the strongest model, which explained about 19% of the variation in age. Removing wing length from this model reduced our predictive capacity to 14% (Fig. 7a, b), confirming that size provides additional descriptive capacity beyond that afforded by the most commonly used entomological indicator, abundance. These observations of field populations serve to verify previously published experimental findings by this study’s authors that found similar results when testing models to predict age at death in a controlled laboratory setting. This previous study found that in *Ae.* *aegypti* reared from eggs collected in Tucson, temperature during development, relative humidity during adulthood, and wing length were the most useful variables for use in the model (but not crowding during development and temperature during adulthood) and predicted 15% of the variation in age at death [69].

As noted in the results, there was an overlap of three days included in the temperature averages generated for the week prior to collection and the developmental period of the youngest age group. Ultimately, the set of variables in the best performing model did not include temperature in the week prior to capture, so there was no overlap in variables in the final model. In contrast, relative humidity in the week prior to capture was significant for predicting age, likely because low humidity is more detrimental to the survival of small vs. large adult mosquitoes because of their increased surface area relative to volume [45–48]. Keeping in mind that nulliparous females are likely to be younger than parous females (females are not immediately ready to take a bloodmeal after emerging), we also found that parous females were significantly larger than nulliparous females (Fig. 4b), indicating that environmental conditions during adulthood caused higher mortality in small females. Furthermore, nulliparous females had a stronger association between size and temperature during development than parous females (Fig. 4). These data show that environmental conditions during adulthood caused increased mortality in small vs. large females, obscuring the relationship between body size and temperature during development in older (parous) females. This insight is useful because it shows that surveillance of body size coupled with measures of age can be used to explain drivers of mortality in adults. Additionally, since only parous females were selected for age grading, it is likely that the benefit of using body size to predict age is underestimated in this study.

Our models in the current study were limited by mortality in the traps, which selected against smaller mosquitoes. This biased the subset of females being tested for age as only females that were collected alive were suitable for processing. Although previous studies suggest that there is no size-associated bias produced by using BG Sentinel traps [74], our findings show that there was a size-associated bias in adult trapping, which should be considered in future studies. It is possible that the increased mortality in small females may have impacted the effect size in one or more cities. Despite this, we were still able to predict a non-trivial amount of variation in age in the Hermosillo population, which could indicate that the real effect size is 19% at a minimum and/or that this model only holds true for mosquitoes beyond a certain size. Although unfortunate for this study, these data are useful for designing future studies in locations at the edge of the geographic range of *Ae.* *aegypti* where aridity is likely to negatively impact survival. Future studies could prioritize trap collections twice per day to minimize size-associated mortality.

We also tested whether using remote weather sensors at each collection site improves data for mosquito surveillance compared to using more readily accessible data such as those made available by the NOAA. We found that, indeed, models using site-specific HOBO data were stronger than those using city-specific NOAA data. However, as noted above, the differences between NOAA and HOBO models for predicting body size were much smaller than the differences between models when predicting age. This is likely due to effects of intra-city variation in environmental factors including temperature during development and relative humidity, which may have impacted adult survival and longevity. This finding supports previous studies that have shown how weather stations do not accurately reflect the microclimates experienced by mosquitoes [75]. Importantly, this finding can be used to justify the additional cost and effort associated with monitoring weather at multiple sites within a city.

It is important to note that using weather data and size measurements requires very little technical expertise, can be measured quickly, and does not require expensive equipment or materials compared to other methods used for characterizing age structure in field mosquitoes. Compared to other methods for determining chronological age in field-collected mosquitoes including characterization of gene transcription profiles [54, 55], analysis of cuticular hydrocarbons using mass chromatography/mass spectrometry [76], or quantification of pteridine fluorescence [77], these benefits strengthen the case for size measurements to be included in surveillance protocols conducted by health departments. The physiological age-determination method of parity assignment via analysis of ovary tracheation is more directly comparable to the method we have tested here of using size measurements to inform models of age structure. Comparing between these two methods, body size data have the advantage of providing continuous vs. categorical data.

## Conclusions

In this study, our model was able to explain 19% of the variation in age in a population of *Ae.* *aegypti* at the edge of (but within) the geographic range of dengue transmission, and 8% of the variation in age in two neighboring populations just beyond the edge of that range. Although in this study wing length was not associated with age and could not serve as its direct proxy, it can potentially serve as an on-the-ground entomological variable that can be collected alongside other transmission-related traits as a part of routine surveillance activities and to inform mathematical models for use in risk-prediction. Longevity and body size have previously been shown to be positively associated [40, 64, 78], with temperature reversing the direction of association [38, 69, 79]. The use of body size in individual-/agent-based mathematical models could be used to parameterize several of the factors driving heterogeneity in vectorial capacity within a city. Furthermore, our findings add to a growing body of evidence that the accuracy of temperature-dependent vector-borne disease model frameworks might be improved by including vector size and local climate data [75]. This article represents an important step towards addressing these gaps and builds upon previous experimental findings that inclusion of wing length data improves estimates of age over using weather data alone [60]. Future research should seek to incorporate mosquito body size in the parameterization of mathematical models to better account for the impacts of environmental conditions on the extrinsic incubation period, dispersal, biting frequency, and survival. More research is needed to validate the results of this work and to improve our understanding of the interactions among weather, eco-physiology of mosquito vectors, and the impacts of these factors on their associated pathogens.

## Supplementary Information

**Additional** **file** **1:** **Figure** **S1.** Distributions of wing length. Stars indicate instances of normal distribution of wing length based on the results of the Shapiro-Wilks test.

**Additional file 2:** **Figure** **S2.** Variation in age among cities.

**Additional file 3:** **Figure** **S3.** Variation in size among cities.

**Additional file 4:** **Table S1**. Collection site locations. **Table S2.** Median collection dates. **Table S3**. Calculated developmental periods. **Table S4**. Average wing lengths and standard deviations, by city. **Table S5**. Factors used in analysis of the response variable, age. Continuous variables were tested in regressions and path analyses. The categorical variables were used for obtaining individual-specific estimates of when the developmental period occurred and for ANOVA tests. For the variable female abundance, the trap count per site is the total from each 4-day monthly sampling period. ^a^One outlier wing length of 1.2 mm was excluded from analysis. (/) No data available.

## Data Availability

The city-specific climate datasets used and analyzed during the current study are available the National Oceanic and Atmospheric Association’s Climate Data Online Search Tool at https://www.ncdc.noaa.gov/cdo-web/search. Other datasets used and/or analyzed during the current study are available from the corresponding author on reasonable request.

## References

[1] Gubler DJ (2012). The economic burden of dengue. Am J Trop Med Hyg.

[2] Halasa YA, Shepard DS, Zeng W (2012). Economic cost of dengue in Puerto Rico. Am J Trop Med Hyg.

[3] WHO. Dengue and severe dengue [Internet]. WHO. 2019. Available from: http://www.who.int/mediacentre/factsheets/fs117/en/

[4] Sharp TM, Margolis HS, Hunsperger E, Tomashek KM, Muñoz-Jordán JL (2014). Sequential episodes of dengue—Puerto Rico, 2005–2010. Am J Trop Med Hyg.

[5] Forshey BM, Reiner RC, Olkowski S, Morrison AC, Espinoza A, Long KC (2016). Incomplete protection against dengue virus type 2 re-infection in Peru. PLoS Negl Trop Dis.

[6] Waggoner JJ, Balmaseda A, Gresh L, Sahoo MK, Montoya M, Wang C (2016). Homotypic dengue virus reinfections in nicaraguan children. J Infect Dis.

[7] Liu-Helmersson J, Stenlund H, Wilder-Smith A, Rocklöv J (2014). Vectorial capacity of *Aedes aegypti*: effects of temperature and implications for global dengue epidemic potential. PLoS ONE.

[8] Pascual M, Ahumada JA, Chaves LF, Rodó X, Bouma M (2006). Malaria resurgence in the East African highlands: temperature trends revisited. Proc Nat Acad Sci..

[9] Patz JA, Reisen WK (2001). Immunology, climate change and vector-borne diseases. Trends Immunol.

[10] Dye C (1986). Vectorial capacity: must we measure all its components?. Parasitol Today..

[11] Ernst KC, Walker KR, Reyes-Castro P, Joy TK, Castro-Luque AL, Diaz-Caravantes RE (2016). Aedes aegypti (Diptera: Culicidae) longevity and differential emergence of dengue fever in two cities in Sonora, Mexico. J Med Entomol..

[12] Walker KR, Williamson D, Carrière Y, Reyes-Castro PA, Haenchen S, Hayden MH (2018). Socioeconomic and human behavioral factors associated with *Aedes aegypti* (Diptera: Culicidae) immature habitat in Tucson, AZ. J Med Entomol..

[13] Li R, Xu L, Bjørnstad ON, Liu K, Song T, Chen A (2019). Climate-driven variation in mosquito density predicts the spatiotemporal dynamics of dengue. Proc Natl Acad Sci USA.

[14] Ng KC, Chaves LF, Tsai KH, Chuang TW (2018). Increased adult aedes aegypti and culex quinquefasciatus (Diptera: Culicidae) abundance in a dengue transmission hotspot, compared to a coldspot, within Kaohsiung city, Taiwan. Insects.

[15] Merrill SA, Ramberg FB, Hagedorn HH (2005). Phylogeography and population structure of *Aedes aegypti* in Arizona. Am J Trop Med Hyg..

[16] Black WC, Bennett KE, Gorrochótegui-Escalante N, Barillas-Mury CV, Fernández-Salas I, Muñoz MDL (2002). Flavivirus susceptibility in *Aedes aegypti*. Arch Med Res.

[17] Chan M, Johansson MA (2012). The incubation periods of Dengue viruses. PLoS ONE.

[18] Padmanabha H, Correa F, Rubio C, Baeza A, Osorio S, Mendez J (2015). Human social behavior and demography drive patterns of fine-scale dengue transmission in endemic areas of colombia. PLoS ONE.

[19] Kamiya T, Greischar MA, Wadhawan K, Gilbert B, Paaijmans K, Mideo N (2020). Temperature-dependent variation in the extrinsic incubation period elevates the risk of vector-borne disease emergence. Epidemics.

[20] Brady OJ, Golding N, Pigott DM, Kraemer MUG, Messina JP, RCR, (2014). Global temperature constraints on *Aedes aegypti* and *Ae. albopictus* persistence and competence for dengue virus transmission. Parasites Vectors..

[21] Araújo MDS, Gil LHS, de Almeida Silva EA (2012). Larval food quantity affects development time, survival and adult biological traits that influence the vectorial capacity of Anopheles darlingi under laboratory conditions. Malar J.

[22] Evans MV, Shiau JC, Solano N, Brindley MA, Drake JM, Murdock CC (2018). Carry-over effects of urban larval environments on the transmission potential of dengue-2 virus. Parasites Vectors.

[23] Bara J, Rapti Z, Cáceres CE, Muturi EJ (2015). Effect of larval competition on extrinsic incubation period and vectorial capacity of *Aedes albopictus* for dengue virus. PLoS ONE.

[24] Scott TW, Amerasinghe P (2000). Longitudinal studies of *Aedes aegypti* (Diptera: Culicidae) in Thailand and Puerto Rico: blood feeding frequency. J Med.

[25] Hatle JD, Paterson CS, Jawaid I, Lentz C, Wells SM, Fronstin RB (2008). Protein accumulation underlying lifespan extension via ovariectomy in grasshoppers is consistent with the disposable soma hypothesis but is not due to dietary restriction. Exp Gerontol..

[26] Riehle MA, Brown MR (1999). Insulin stimulates ecdysteroid production through a conserved signaling cascade in the mosquito *Aedes aegypti*. Insect Biochem Mol Biol.

[27] Riehle MA, Brown MR (2002). Insulin receptor expression during development and a reproductive cycle in the ovary of the mosquito *Aedes aegypti*. Cell Tissue Res.

[28] Arik AJ, Rasgon JL, Quicke KM, Riehle MA (2009). Manipulating insulin signaling to enhance mosquito reproduction. BMC Physiol.

[29] Arik AJ, Hun LV, Quicke K, Piatt M, Ziegler R, Scaraf PY (2016). Increased Akt signaling in the mosquito fat body increases adult survivorship. FASEB J.

[30] Clifton M, Noriega FG (2012). The fate of follicles after a blood meal is dependent on previtellogenic nutrition and juvenile hormone in *Aedes aegypti*. J Insect Physiol.

[31] Noriega FG (2004). Nutritional regulation of JH synthesis: a mechanism to control reproductive maturation in mosquitoes?. Insect Biochem Mol Biol.

[32] Davidowitz G (2016). Endocrine proxies can simplify endocrine complexity to enable evolutionary prediction. Integr Comp Biol..

[33] Zeller M, Koella JC (2016). Effects of food variability on growth and reproduction of *Aedes*
*aegypti*. Ecol Evol..

[34] Huxley PJ, Murray KA, Pawar S, Cator LJ (2022). Competition and resource depletion shape the thermal response of population fitness in *Aedes aegypti*. Commun Biol Nat Res.

[35] Huxley PJ, Murray KA, Pawar S, Cator LJ (2021). The effect of resource limitation on the temperature dependence of mosquito population fitness. Proc R Soc B Biol Sci.

[36] Forster J. Exploring the mechanism of how ectotherms change size with changing temperature. 2012;209.

[37] Savage VM, Gillooly JF, Brown JH, West GB, Charnov EL (2004). Effects of body size and temperature on population growth. Am Nat.

[38] Norry FM, Loeschcke V (2002). Temperature-induced shifts in associations of longevity with body size in Drosophila Melanogaster. Evolution (N Y)..

[39] Briegel H (1990). Metabolic relationship between female body size, reserves, and fecundity of *Aedes aegypti*. J Insect Physiol.

[40] Mogi M, Miyagi I, Syafruddin AK (1996). Inter- and intraspecific variation in resistance to desiccation by adult Aedes (Stegomyia) spp. (Diptera: Culicidae) from Indonesia. J Med Entomol..

[41] Davidowitz G (2008). Population and environmental effects on the size-fecundity relationship in a common grasshopper across an aridity gradient. J Orthoptera Res..

[42] Joy TK, Arik AJ, Corby-Harris V, Johnson AA, Riehle MA (2010). The impact of larval and adult dietary restriction on lifespan, reproduction and growth in the mosquito *Aedes aegypti*. Exp Gerontol.

[43] Helinski MEH, Harrington LC (2011). Male mating history and body size influence female fecundity and longevity of the dengue vector *Aedes aegypti*. J Med Entomol.

[44] Barreaux AMG, Stone CM, Barreaux P, Koella JC (2018). The relationship between size and longevity of the malaria vector *Anopheles gambiae* (ss) depends on the larval environment. Parasites Vectors.

[45] Gibbs A (1997). Physiological mechanisms of evolved desiccation resistance in Drosophila Melanogaster. J Exp Biol.

[46] Gibbs AG, Fukuzato F, Matzkin LM (2003). Evolution of water conservation mechanisms in Drosophila. J Exp Biol.

[47] Fouet C, Gray E, Besansky NJ, Costantini C (2012). Adaptation to aridity in the malaria mosquito *Anopheles gambiae*: chromosomal inversion polymorphism and body size influence resistance to desiccation. PLoS ONE.

[48] Hercus MJ, Hoffmann AA (1999). Desiccation resistance in interspecific drosophila crosses: genetic interactions and trait correlations. Genetics.

[49] Schmidt CA, Comeau G, Monaghan AJ, Williamson DJ, Ernst KC (2018). Effects of desiccation stress on adult female longevity in *Aedes aegypti* and *Ae. albopictus* (Diptera: Culicidae): results of a systematic review and pooled survival analysis. Parasit Vectors..

[50] Reiskind MH, Lounibos LP (2009). Effects of intraspecific larval competition on adult longevity in the mosquitoes *Aedes aegypti* and *Aedes albopictus*. Med Vet Entomol.

[51] Peter Marian M, Christopher MS, Selvaraj AM, Pandian TJ (1983). Studies on predation of the mosquito *Culex **fatigans* by *Rana **tigrina* tadpoles. Hydrobiologia.

[52] Gonsalves L, Bicknell B, Law B, Webb C, Monamy V (2013). Mosquito consumption by insectivorous bats: does size matter?. PLoS ONE.

[53] Maciel-De-Freitas R, Codeço CT, Lourenço-De-Oliveira R (2007). Body size-associated survival and dispersal rates of *Aedes aegypti* in Rio de Janeiro. Med Vet Entomol.

[54] Joy TK, Gutierrez EHJ, Ernst K, Walker KR, Carriere Y, Torabi M (2012). Aging field collected *Aedes aegypti* to determine their capacity for dengue transmission in the southwestern United States. PLoS ONE.

[55] Cook PE, Hugo LE, Iturbe-Ormaetxe I, Williams CR, Chenoweth SF, Ritchie SA (2006). The use of transcriptional profiles to predict adult mosquito age under field conditions. Proc Natl Acad Sci USA.

[56] Scott TW, Morrison A, Lorenz L (2000). Longitudinal studies of *Aedes aegypti* (Diptera: Culicidae ) in Thailand and Puerto Rico: population dynamics. J Med.

[57] Krockel U, Rose A, Eiras AEGM (2006). New tools for surveillance of adult yellow fever mosquitoes: comparison of trap catches with human landing rates in an urban environment. J Am Mosq Control Assoc.

[58] Maciel-de-Freitas R, Eiras ÁE, Lourenço-de-Oliveira R (2006). Field evaluation of effectiveness of the BG-Sentinel, a new trap for capturing adult *Aedes aegypti* (Diptera: Culicidae). Mem Inst Oswaldo Cruz.

[59] Williams CR, Long SA, Russell RC, Ritchie SA (2006). Field efficacy of the BG-sentinel compared with CDC backpack aspirators and CO_2_-baited EVS traps for collection of adult *Aedes aegypti* in Cairns, Queensland, Australia. J Am Mosq Control Assoc..

[60] Ball TS, Ritchie SR (2010). Evaluation of BG-sentinel trap trapping efficacy for *Aedes aegypti* (Diptera: Culicidae) in a visually competitive environment. J Med Entomol.

[61] Salazar FV, Achee NL, Grieco JP, Prabaripai A, Ojo TA, Eisen L (2013). Effect of *Aedes aegypti* exposure to spatial repellent chemicals on BG-Sentinel TM trap catches. Parasit Vectors.

[62] Detinova TS (1962). Age grouping methods in diptera of medical importance: with special reference to some vectors of malaria. World Heal Organ Monogr Ser.

[63] Tyndale-Biscoe M (1984). Age-grading methods in adult insects: a review. Bull Entomol Res.

[64] Nasci RS (1986). The size of emerging and host-seeking *Aedes Aegypti* and the relation of size to blood-feeding success in the field. J Am Mosq Control Assoc.

[65] Nasci RS (1991). Influence of larval and adult nutrition on biting persistence in *Aedes aegypti* (Diptera: Culicidae). J Med Entomol.

[66] Davis EE (1984). Development of lactic acid-receptor sensitivity and host-seeking behaviour in newly emerged female *Aedes aegypti* mosquitoes. J Insect Physiol Pergamon.

[67] Joy T, Chen M, Arnbrister J, Williamson D, Li S, Nair S (2022). Assessing Near-Infrared Spectroscopy (NIRS) for Evaluation of Aedes aegypti Population Age Structure. Insects MDPI.

[68] Van Handel E, Day FJ (1989). Correlation between wing length and protein content of mosquitoes. J Am Mosq Control.

[69] Jeffrey Gutiérrez EH, Walker KR, Ernst KC, Riehle MA, Davidowitz G (2020). Size as a proxy for survival in *Aedes aegypti* (Diptera: Culicidae) Mosquitoes. J Med Entomol..

[70] Morales Vargas RE, Ya-Umphan P, Phumala-Morales N, Komalamisra N, Dujardin J-P (2010). Climate associated size and shape changes in *Aedes aegypti* (Diptera: Culicidae) populations from Thailand. Infect Genet Evol..

[71] The R Foundation. R: The R Project for Statistical Computing [Internet]. 2019. Available from: https://www.r-project.org/

[72] SAS Institute. JMP Statistical Software from SAS [Internet]. 2019. Available from: https://www.jmp.com/en_us/home.html

[73] McDonald RP, Ho M-HR (2002). Principles and practice in reporting structural equation analyses. Psychol Methods.

[74] Ball TS, Ritchie SR (2010). Sampling biases of the BG-sentinel trap with respect to physiology, age, and body size of adult *Aedes aegypti* (Diptera: Culicidae). J Med Entomol..

[75] Murdock CC, Evans MV, McClanahan TD, Miazgowicz KL, Tesla B (2017). Fine-scale variation in microclimate across an urban landscape shapes variation in mosquito population dynamics and the potential of *Aedes albopictus* to transmit arboviral disease. PLoS Negl Trop Dis..

[76] Desena ML, Edman JD, Clark JM, Symington SB, Scott TW (1999). *Aedes aegypti* (Diptera: Culicidae) age determination by cuticular hydrocarbon analysis of female legs. J Med Entomol.

[77] Wu D, Lehane MJ (1999). Pteridine fluorescence for age determination of Anopheles mosquitoes. Med Vet Entomol.

[78] Alto BW, Bettinardi DJ, Ortiz S (2015). Interspecific larval competition differentially impacts adult survival in dengue vectors. J Med Entomol..

[79] Mourya DT, Yadav P, Mishra AC (2004). Effect of temperature stress on immature stages and susceptibility of *Aedes aegypti* mosquitoes to chikungunya virus. Am J Trop Med Hyg.

