# Supplementary figures and images for: Using body size as an indicator for age structure in field populations of Aedes aegypti (Diptera: Culicidae)
Source: Parasit Vectors. 2022 Dec 22;15:483. doi: 10.1186/s13071-022-05605-z (PMC9773510; doi:10.1186/s13071-022-05605-z)

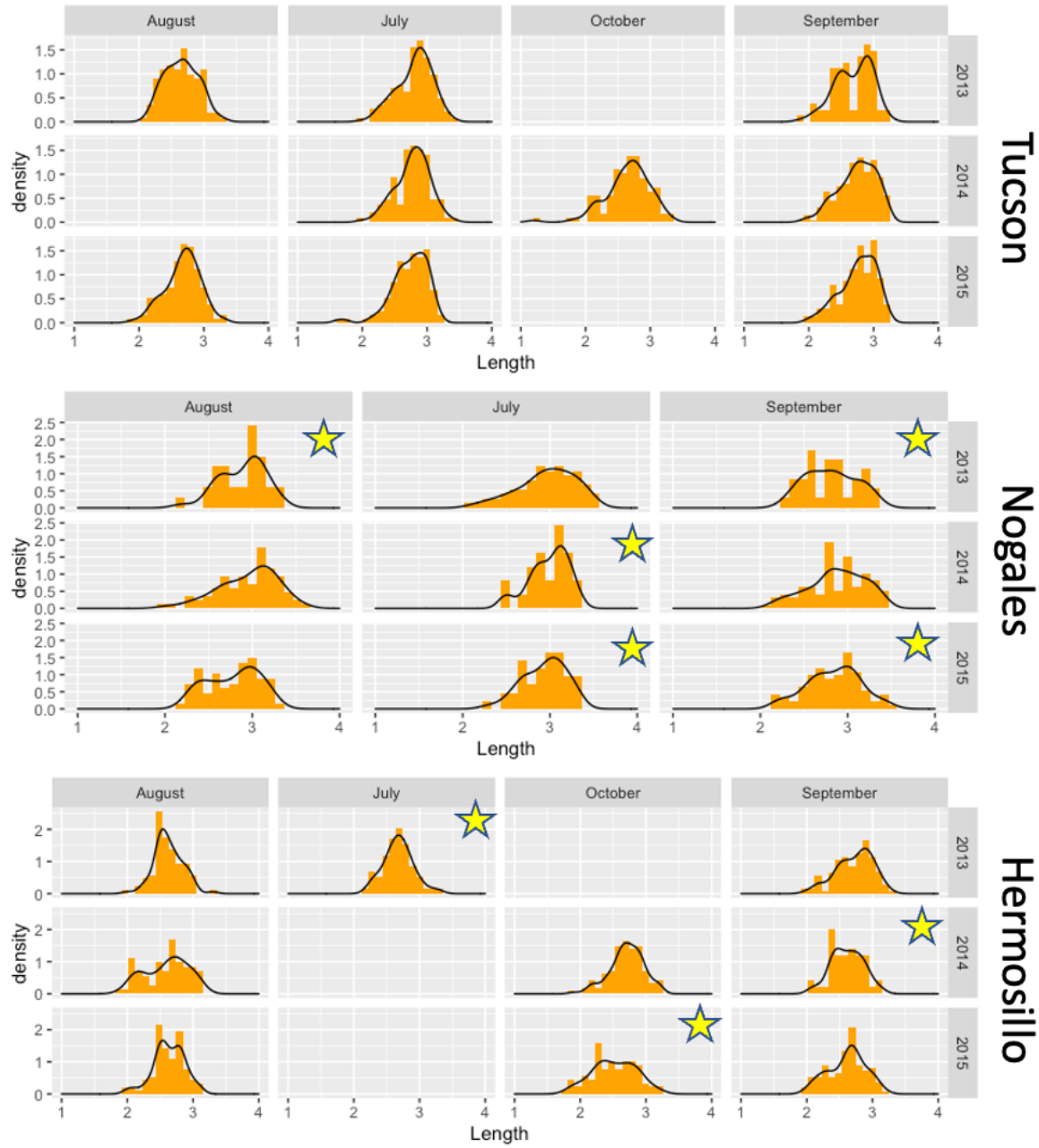

Supplement: Supplementary file 1 — Additional file 1: Figure S1. Distributions of wing length. Stars indicate instances of normal distribution of wing length based on the results of the Shapiro-Wilks test. [file 13071_2022_5605_MOESM1_ESM.pdf]

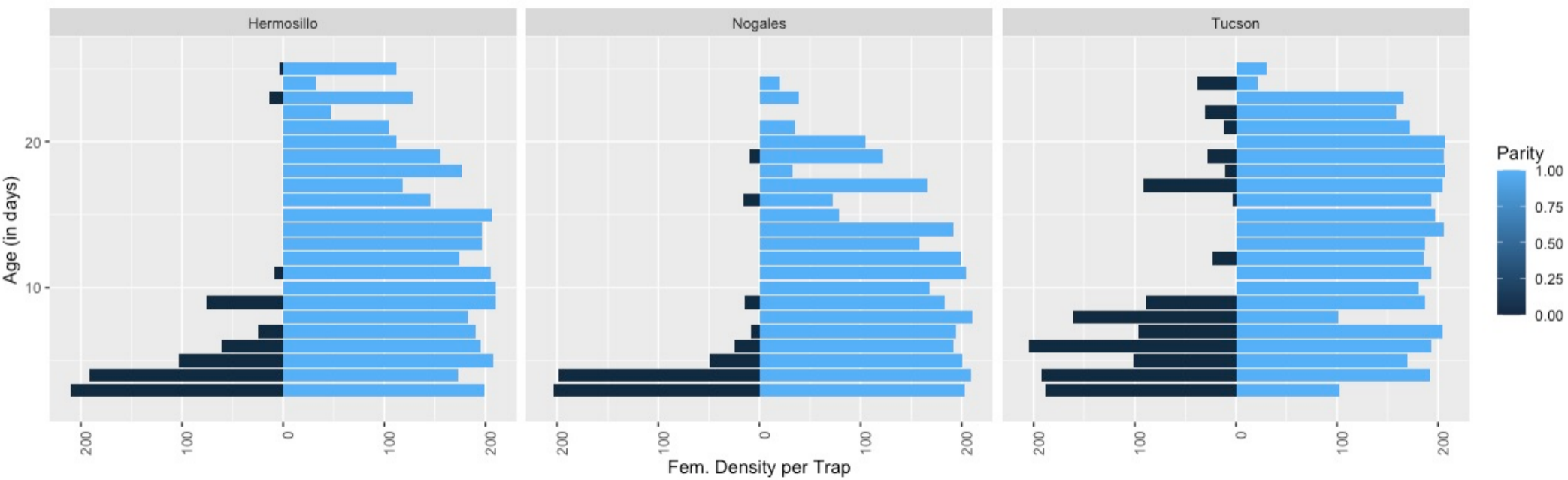

Supplement: Supplementary file 2 — Additional file 2: Figure S2. Variation in age among cities. [file 13071_2022_5605_MOESM2_ESM.pdf]

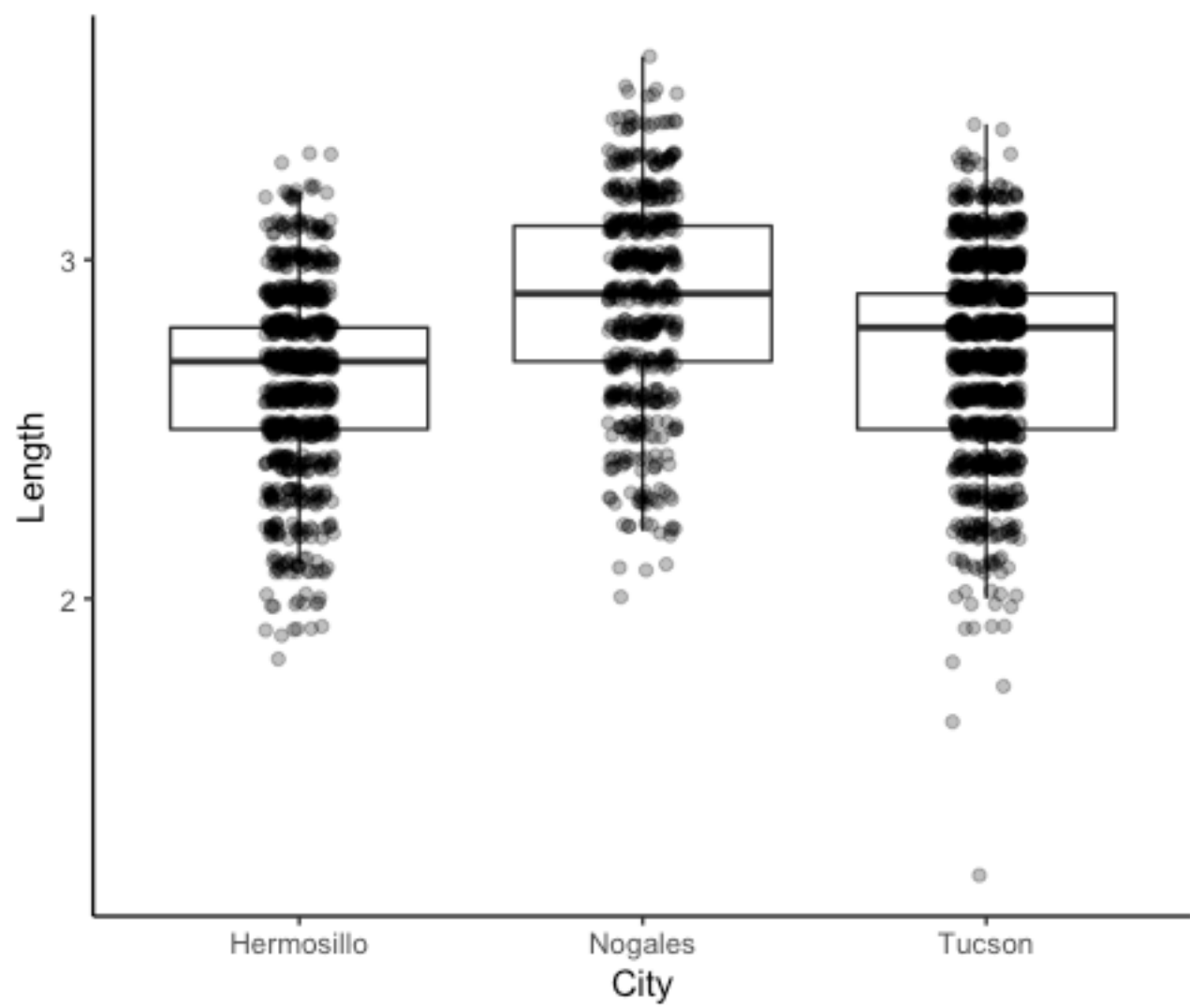

Supplement: Supplementary file 3 — Additional file 3: Figure S3. Variation in size among cities. [file 13071_2022_5605_MOESM3_ESM.pdf]
