# Supplementary material for: Using body size as an indicator for age structure in field populations of Aedes aegypti (Diptera: Culicidae)
Source: Parasit Vectors. 2022 Dec 22;15:483. doi: 10.1186/s13071-022-05605-z (PMC9773510; doi:10.1186/s13071-022-05605-z)
Supplement: Supplementary file 4 — Additional file 4: Table S1. Collection site locations. Table S2. Median collection dates. Table S3. Calculated developmental periods. Table S4. Average wing lengths and standard deviations, by city. Table S5. Factors used in analysis of the response variable, age. Continuous variables were tested in regressions and path analyses. The categorical variables were used for obtaining individual-specific estimates of when the developmental period occurred and for ANOVA tests. For the variable female abundance, the trap count per site is the total from each 4-day monthly sampling period. aOne outlier wing length of 1.2 mm was excluded from analysis. (/) No data available. [file 13071_2022_5605_MOESM4_ESM.docx]

**Table S1: Collection site locations.**

| **City** | **Site** | **Latitude** | **Longitude** |
| --- | --- | --- | --- |
| Hermosillo | 1 | 29.08268637 | -110.9556745 |
|  | 2 | 29.12871490 | -110.9547237 |
|  | 3 | 29.13416320 | -110.9654744 |
|  | 4 | 29.12245072 | -110.9720153 |
|  | 5 | 29.09152731 | -110.9815320 |
|  | 6 | 29.07245979 | -110.9721396 |
|  | 7 | 29.07992685 | -110.9857602 |
|  | 8 | 29.10399660 | -111.0019631 |
|  | 9 | 29.10079634 | -111.0141063 |
|  | 10 | 29.11775670 | -111.0198957 |
|  | 11 | 29.10761671 | -111.0431564 |
|  | 12 | 29.05892086 | -110.9570923 |
|  | 13 | 29.03327954 | -110.9511223 |
|  | 14 | 29.05154527 | -110.9784334 |
|  | 15 | 29.01640000 | -110.9426000 |
|  | 16 | 29.10780000 | -110.9504000 |
|  | 17 | 29.14258086 | -110.9596319 |
| Nogales | 1 | 31.33108404 | -110.9350266 |
|  | 2 | 31.32501188 | -110.9405280 |
|  | 3 | 31.31455915 | -110.9312902 |
|  | 4 | 31.29938871 | -110.9324453 |
|  | 5 | 31.29254535 | -110.9190702 |
|  | 6 | 31.29110934 | -110.9328825 |
|  | 7 | 31.27021801 | -110.9258818 |
|  | 8 | 31.30185995 | -110.9015727 |
|  | 9 | 31.29253138 | -110.9734403 |
|  | 10 | 31.26590555 | -110.9653378 |
|  | 11 | 31.26032352 | -110.9553957 |
|  | 12 | 31.28442673 | -110.9634350 |
|  | 13 | 31.27715269 | -110.9537219 |
|  | 14 | 31.30334091 | -110.9514452 |
|  | 15 | 31.31134307 | -110.9501639 |
| Tucson | 2 | 32.26291400 | -110.9312340 |
|  | 5 | 32.25332100 | -110.9281650 |
|  | 8 | 32.23882200 | -110.9333530 |
|  | 13 | 32.22964700 | -110.9226210 |
|  | 14 | 32.21849100 | -110.9280050 |
|  | 16 | 32.24414100 | -110.9042530 |
|  | 17 | 32.23904500 | -110.8887900 |
|  | 19 | 32.22676600 | -110.9013900 |
|  | 24 | 32.21099500 | -110.9034490 |
|  | 25 | 32.21249600 | -110.8845140 |
|  | 27 | 32.20269300 | -110.8430220 |
|  | 28 | 32.23479400 | -110.9901250 |
|  | 30 | 32.21391600 | -110.9755490 |
|  | 33 | 32.20025200 | -110.9217470 |
|  | 36 | 32.16427400 | -110.9492770 |
|  | 39 | 32.14625500 | -110.9503680 |

**Table S2: Median collection dates.**

| **City** | **Year** | **Median Collection Date** | | |
| --- | --- | --- | --- | --- |
|  |  | **Round 1** | **Round 2** | **Round 3** |
| Tucson | 2013 | 7/24/13 | 8/28/13 | 9/24/13 |
|  | 2014 | 7/30/14 | 9/4/14 | 10/1/14 |
|  | 2015 | 7/22/15 | 8/19/15 | 9/16/15 |
| Nogales | 2013 | 7/24/13 | 8/21/13 | 9/16/13 |
|  | 2014 | 7/30/14 | 8/27/14 | 9/24/14 |
|  | 2015 | 7/29/15 | 8/27/15 | 9/24/15 |
| Hermosillo | 2013 | 7/18/13 | 8/18/13 | 9/12/13 |
|  | 2014 | 8/13/14 | 9/10/14 | 10/8/14 |
|  | 2015 | 8/12/15 | 9/9/15 | 10/7/15 |

**Table S3: Calculated developmental periods.**

| **City** | **Year** | **Collection** | **Estimated Developmental Period** | | |
| --- | --- | --- | --- | --- | --- |
|  |  |  | **Age 1** | **Age 2** | **Age 3** |
| Tucson | 2013 | Round 1 | 7/14/13 - 7/20/13* | 7/7/13 - 7/13/13* | 7/2/13 - 7/8/13* |
|  |  | Round 2 | 8/19/13 - 8/25/13 | 8/12/13 - 8/18/13 | 8/8/13 - 8/14/13 |
|  |  | Round 3 | 9/15/13 - 9/21/13 | 9/8/13 - 9/14/13 | 9/4/13 - 9/10/13 |
|  | 2014 | Round 1 | 7/21/14 - 7/27/14 | 7/14/14 - 7/20/14 | 7/9/14 - 7/15/14 |
|  |  | Round 2 | 8/26/14 - 9/1/14 | 8/19/14 - 8/25/14 | 8/14/14 - 8/20/14 |
|  |  | Round 3 | 9/22/14 - 9/28/14 | 9/15/14 - 9/21/14 | 9/10/14 - 9/16/14 |
|  | 2015 | Round 1 | 7/13/15 - 7/19/15 | 7/6/15 - 7/12/15 | 7/2/15 - 7/8/15 |
|  |  | Round 2 | 8/10/15 - 8/16/15 | 8/3/15 - 8/9/15 | 7/29/15 - 8/5/15 |
|  |  | Round 3 | 9/7/15 - 9/13/15 | 8/31/15 - 9/6/15 | 8/27/15 - 9/2/15 |
| Nogales | 2013 | Round 1 | 7/14/13 - 7/20/13* | 7/7/13 - 7/13/13* | 7/2/13 - 7/8/13* |
|  |  | Round 2 | 8/12/13 - 8/18/13** | 8/5/13 - 8/11/13* | 7/31/13 - 8/6/13* |
|  |  | Round 3 | 9/7/13 - 9/13/13 | 8/31/13 - 9/6/13 | 8/26/13 - 9/1/13 |
|  | 2014 | Round 1 | 7/21/14 - 7/27/14 | 7/14/14 - 7/20/14 | 7/9/14 - 7/15/14 |
|  |  | Round 2 | 8/18/14 - 8/24/14 | 8/11/14 - 8/17/14 | 8/6/14 - 8/12/14 |
|  |  | Round 3 | 9/15/14 - 9/21/14 | 9/8/14 - 9/14/14 | 9/3/14 - 9/9/14 |
|  | 2015 | Round 1 | 7/20/15 -7/26/15 | 7/13/15 -7/19/15 | 7/9/15 -7/15/15 |
|  |  | Round 2 | 8/18/15 - 8/24/15 | 8/11/15 - 8/17/15 | 8/7/15 - 8/13/15 |
|  |  | Round 3 | 9/15/15 - 9/21/15 | 9/8/15 - 9/14/15 | 9/4/15 - 9/10/15 |
| Hermosillo | 2013 | Round 1 | 7/8/13 - 7/14/13* | 7/1/13 - 7/7/13* | 6/26/13 - 7/2/13* |
|  |  | Round 2 | 8/9/13 - 8/15/13 | 8/1/13 - 8/7/13* | 7/27/13 - 8/2/13* |
|  |  | Round 3 | 9/3/13 - 9/9/13 | 8/27/13 - 9/2/13 | 8/23/13 - 8/29/13 |
|  | 2014 | Round 1 | 8/4/14 - 8/10/14 | 7/28/14 - 8/3/14 | 7/23/14 - 7/29/14 |
|  |  | Round 2 | 9/1/14 - 9/7/14 | 8/25/14 - 8/31/14 | 8/19/14 - 8/26/14 |
|  |  | Round 3 | 9/29/14 - 10/5/14 | 9/22/14 - 9/28/14 | 9/17/14 - 9/23/14 |
|  | 2015 | Round 1 | 8/2/15 - 8/9/15* | 7/26/15 - 8/2/15* | 7/21/15 - 7/28/15* |
|  |  | Round 2 | 8/30/15 - 9/6/15* | 8/23/15 - 8/30/15* | 8/18/15 - 8/25/15* |
|  |  | Round 3 | 9/27/15 - 10/4/15* | 9/20/15 - 9/27/15* | 9/15/15 - 9/22/15* |

**Table S4: Average wing lengths and standard deviations, by city.**

| **City** | **2013** | | | **2013 Avg.** | **2014** | | | | **2014 Avg.** | **2015** | | | | **2015 Avg.** | **Avg. All Years** |
| --- | --- | --- | --- | --- | --- | --- | --- | --- | --- | --- | --- | --- | --- | --- | --- |
|  | **July** | **August** | **September** |  | **July** | **August** | **September** | **October** |  | **July** | **August** | **September** | **October** |  |  |
| Hermosillo | 2.68, 0.23 | 2.63, 0.22 | 2.73, 0.28 | 2.69, 0.26 | / | 2.60, 0.33 | 2.62, 0.24 | 2.71, 0.26 | 2.65, 0.28 | / | 2.63, 0.24 | 2.61, 0.28 | 2.52, 0.33 | 2.59, 0.29 | 2.64, 0.28 |
| Nogales | 2.97, 0.33 | 2.88, 0.26 | 2.81, 0.29 | 2.91, 0.31 | 2.99, 0.22 | 2.95, 0.34 | 2.88, 0.32 | / | 2.92, 0.32 | 2.93, 0.25 | 2.78, 0.30 | 2.84, 0.31 | / | 2.84, 0.30 | 2.89, 0.31 |
| Tucson | 2.81, 0.28 | 2.65, 0.26 | 2.69, 0.29 | 2.73, 0.28 | 2.78, 0.26 | / | 2.75, 0.29 | 2.65, 0.34 | 2.73, 0.30 | 2.74, 0.28 | 2.67, 0.27 | 2.76, 0.28 | / | 2.73, 0.28 | 2.73, 0.29 |

**Table S5:** **Factors used in analysis of the response variable, age.**

| Explanatory variables | Sample size | Mean | SD | SE | Variable type | Range |
| --- | --- | --- | --- | --- | --- | --- |
| Temperature during development (HOBO) | 1,442 | 28.6 | 3.1 | 0.1 | Continuous | 21.3-36.5℃ |
| Wing length | 2,443 | 2.7 | 0.3 | 0.0 | Continuous | 1.6^a^-3.6mm |
| Female abundance (trap count per site) | 3,027 | 27.8 | 25.6 | 0.5 | Continuous | 1-202 |
| Relative humidity 1 week prior to capture (HOBO) | 2,354 | 47.8 | 14.9 | 0.3 | Continuous | 26.5-91.5% |
| Temperature 1 week prior to capture (HOBO) | 2,355 | 28.4 | 3.1 | 0.1 | Continuous | 21.4-34.3 |
| Age (used in regressions) | 1,936 | 8.1 days | 5.2 | 0.1 | Continuous | 3-26 |
| Age (used for estimating developmental periods) | 1,936 | / | / | / | Categorical | 1-3 |
| Parity | 2,909 | / | / | / | Categorical | 0-1 |
